# The Impact of Cardiac Radiation Dosimetry on Survival After Radiation Therapy for Non-Small Cell Lung Cancer

**DOI:** 10.1016/j.ijrobp.2017.04.026

**Published:** 2017-09-01

**Authors:** S. Vivekanandan, D.B. Landau, N. Counsell, D.R. Warren, A. Khwanda, S.D. Rosen, E. Parsons, Y. Ngai, L. Farrelly, L. Hughes, M.A. Hawkins, J.D. Fenwick

**Affiliations:** ∗ Department of Oncology and CRUK MRC, Oxford Institute for Radiation Oncology, Gray Laboratories, University of Oxford, Oxford, UK; † Department of Oncology, Guy's & St. Thomas' NHS Trust, King's College London, UK; ‡ Cancer Research UK & UCL Cancer Trials Centre Cancer Institute, University College London, London, UK; § Department of Cardiology, Ealing Hospital and Imperial College London, UK; ‖ Department of Cardiology, Ealing and Royal Brompton Hospitals & Imperial College, London, UK; ¶ Radiotherapy Trials Quality Assurance, Mount Vernon Hospital, Middlesex, UK; # Department of Molecular and Clinical Cancer Medicine, Institute of Translational Medicine, University of Liverpool, Liverpool, UK; ∗∗ Department of Physics, Clatterbridge Cancer Centre, Wirral, UK

## Abstract

**Purpose:**

The heart receives high radiation doses during radiation therapy of advanced-stage lung cancer. We have explored associations between overall survival, cardiac radiation doses, and electrocardiographic (ECG) changes in patients treated in IDEAL-CRT, a trial of isotoxically escalated concurrent chemoradiation delivering tumor doses of 63 to 73 Gy.

**Methods and Materials:**

Dosimetric and survival data were analyzed for 78 patients. The whole heart, pericardium, AV node, and walls of left and right atria (LA/RA-Wall) and ventricles (LV/RV-Wall) were outlined on radiation therapy planning scans, and differential dose-volume histograms (dDVHs) were calculated. For each structure, dDVHs were approximated using the average dDVH and the 10 highest-ranked structure-specific principal components (PCs). ECGs at baseline and 6 months after radiation therapy were analyzed for 53 patients, dichotomizing patients according to presence or absence of “any ECG change” (conduction or ischemic/pericarditis-like change). All-cause death rate (DR) was analyzed from the start of treatment using Cox regression.

**Results:**

38% of patients had ECG changes at 6 months. On univariable analysis, higher scores for LA-Wall-PC6, Heart-PC6, “any ECG change,” and larger planning target volume (PTV) were significantly associated with higher DR (*P*=.003, .009, .029, and .037, respectively). Heart-PC6 and LA-Wall-PC6 represent larger volumes of whole heart and left atrial wall receiving 63 to 69 Gy. Cardiac doses ≥63 Gy were concentrated in the LA-Wall, and consequently Heart-PC6 was highly correlated with LA-Wall-PC6. “Any ECG change,” LA-Wall-PC6 scores, and PTV size were retained in the multivariable model.

**Conclusions:**

We found associations between higher DR and conduction or ischemic/pericarditis-like changes on ECG at 6 months, and between higher DR and higher Heart-PC6 or LA-Wall-PC6 scores, which are closely related to heart or left atrial wall volumes receiving 63 to 69 Gy in this small cohort of patients.

**Summary**

We explored associations between all-cause death rate (DR), cardiac radiation doses, and electrocardiographic changes in 78 patients with locally advanced non-small cell lung cancer treated in IDEAL-CRT, a trial of isotoxically escalated concurrent CRT. We found evidence of associations between higher DR and conduction or ischemic/pericarditis-like changes on ECG at 6 months, and between higher DR and higher heart or left atrial wall volumes receiving 63 to 69 Gy.

## Introduction

Definitive chemoradiation (CRT) is the standard of care for locally advanced non-small cell lung cancer (NSCLC) (1). For more than 30 years the accepted radiation therapy dose was 60 to 63 Gy in 1.8- to 2.0-Gy fractions, established by Radiation Therapy Oncology Group trial RTOG-7301 2, 3. Overall survival (OS) is poor at these dose levels, with high local failure rates stimulating interest in dose escalation. Outcomes modeling suggests a tumor dose response (4), and results from early-phase studies indicated that concurrent CRT might be safe up to 74 Gy 5, 6, 7, 8, 9, 10, 11, 12. However, the RTOG-0617 phase 3 trial of dose escalation has reported a significantly lower OS for 74 Gy than for 60 Gy in daily 2-Gy fractions, triggering efforts to identify reasons for the reduced survival (13). An RTOG-0617 analysis found negative associations between OS and high heart volumes receiving more than 5 Gy or 40 Gy 13, 14. A recent meta-analysis of randomized trials in NSCLC found that for concurrent CRT treatments, higher radiation doses result in poorer OS, possibly partly because of higher levels of toxicity in the presence of concurrent chemotherapy (15).

Radiation-induced heart disease (RIHD) has been associated with poorer long-term OS in breast and lymphoma survivors 16, 17. For locally advanced NSCLC patients, the impact of cardiac irradiation on OS has not been well characterized because RIHD latency was thought to be longer than the typical OS. However, NSCLC patients are generally older than breast cancer or lymphoma patients, have more underlying cardiopulmonary conditions and common risk factors for ischemic heart disease, and receive higher target radiation doses. Interest in RIHD after NSCLC radiation therapy has therefore increased with the emergence of evidence suggesting that RIHD affects OS earlier than was previously thought 18, 19.

In this post-hoc analysis of the prospective data from IDEAL-CRT (20), we aimed to identify the impact of cardiac irradiation on the all-cause death rate (DR) using a dose-volume histogram-wide analysis approach based on principal components analysis (PCA). We further attempted to localize to specific cardiac substructures the associations seen between whole-heart dosimetry and DR, because whole heart avoidance may not be feasible in clinical practice. To explore pathophysiologic connections between cardiac irradiation and survival, we evaluated electrocardiogram (ECG) changes after irradiation.

## Methods and Materials

### Population

Data were analyzed for 78 of all 82 patients with stage IIB/III NSCLC who were recruited and treated in IDEAL-CRT, a phase 1/2 trial of isotoxically escalated CRT, which delivered 63 to 73 Gy in 30 fractions over 6 weeks, concurrently with 2 cycles of vinorelbine and cisplatin. Four patients were excluded, 1 because of corrupted radiation therapy planning archival records, 2 because of replanning during treatment, and 1 because of treatment termination after toxicity after 5 fractions resulting in very low heart doses.

### ECG scoring

The ECGs obtained at baseline and 6 months after CRT were assessed by 2 cardiologists (S.R. and A.K.) in a blinded manner for the following characteristics: normal ECG, new rhythm changes compared with baseline (sinus tachycardia/bradycardia, conduction abnormalities such as bundle branch block, atrial fibrillation), and ischemic or pericarditis-like changes (new or worsening ST and T wave changes compared with baseline). ECG changes between the 2 time points were then scored dichotomously as present or absent (“any ECG change”).

### Processing of radiation therapy dosimetric data

Treatment plans were imported into the open-source Computational Environment for Radiotherapy Research (CERR) software written in MATLAB R2012b (Mathworks, Inc, Natick, MA). Whole-heart contours delineated by physicians at participating centers were checked by DL and SV. The pericardium, right atrium (RA), right ventricle (RV), left atrium (LA), left ventricle (LV), and AV node (AVN) were delineated as individual substructures by SV using a modified version of a validated cardiac atlas (21). The pericardium was defined as a rim volume lying 5 mm or less beyond the heart, and the outlined AVN was expanded by 3 mm superiorly-inferiorly to account for delineation variability. RA-Wall, RV-Wall, LA-Wall, and LV-Wall were defined as wall regions lying 5 mm or less within the RA, RV, LA, and LV contours, respectively.

Differential dose-volume histograms (dDVH) with 1-Gy dose-bins were constructed for the whole heart and all substructures and were exported to SPSS.23 (IBM Corp., Armonk, NY) and R 3.2.3 (R Foundation, Vienna, Austria) for further processing. Separate principal components analyses (PCA) were carried out for the whole heart and for each cardiac substructure, representing patients' dDVHs as linear sums of structure-specific orthogonal principal components (PCs) and the population-averaged dDVH (Appendix A; available online at www.redjournal.org) 22, 23, 24, 25, 26. The scores of the PCs in these linear sums reflect the degrees to which they are present in each patient's dDVH. For each cardiac structure, PCA allowed the highly correlated dose distributions of the whole patient group to be efficiently approximated by a truncated set of PCs, specifically the 10 highest-ranked PCs obtained from PCA for that specific structure. Scores of the initially obtained PCs were uncorrelated across the patient group, each PC describing a unique and independent portion of the dosimetric variability. However, these PCs contained numerous peaks, obscuring their physical interpretation. We therefore used varimax rotation to simplify the PC structure (27), applying an orthogonal rotation to the truncated set of PCs. The rotated PCs are more easily interpretable, mostly having nonnegligible amplitudes across only narrow dose regions, although their scores are no longer orthogonal.

### Statistical analysis

Univariable and multivariable analyses (UVA/MVA) of hazard ratios (HRs) for the all-cause death rate (DR) (measured from the start of treatment) were performed with Cox proportional hazards regression. Factors analyzed for association with DR were as follows: clinical (patient and treatment) characteristics, ECG changes, and scores of the 10 highest-ranked whole-heart dosimetry PCs. Relevant clinical factors, ECG changes, and PCs having *P* values ≤.20 on UVA were initially included in multivariable models, and stepwise bidirectional variable elimination (28) was performed to find the model with the lowest Akaike information criterion (AIC) score (29).

The false-discovery rate for significant associations after multiple hypothesis testing was controlled using the Benjamini-Hochberg step-up procedure to identify positive discoveries (Appendix B; available online at www.redjournal.org). The predictive abilities of multivariable models were characterized using Harrell's concordance (C) statistic 30, 31. The C statistic of a model is 1.0 if for all possible pairs of patients, it correctly predicts which patient has the longer survival. A value of 0.5 indicates a model performance no better than chance.

Associations between dichotomized ECG changes and the scores of PCs significantly associated with DR on MVA were tested using logistic regression.

Two approaches were used to further localize the associations seen between DR and whole-heart dosimetry to specific cardiac substructures. The first involved UVA/MVA of associations between DR and the scores of individual cardiac substructure PCs (ss-PCs). To avoid excessive multiple testing, we studied only ss-PCs closely aligned with “heart-PC_max_,” the whole-heart PC most strongly associated with DR, using a normalized dot product (NDP) criterion described in Appendix C (available online at www.redjournal.org) to assess closeness of alignment.

The second approach was a graphic interrogation of the shapes of the heart dose distributions of all 78 patients, mapped to a single reference geometry using an in-house MATLAB program (described in detail in Appendix D; available online at www.redjournal.org). Binary masks were created from each patient's whole heart and left and right atria and ventricles, and registered to the corresponding masks of a reference patient by affine transformation. 3D dose distributions were mapped to the reference geometry through the same affine transformations, and a threshold was applied to identify the region where dose was associated with the peak in heart-PCmax (as quantified in the Results section) for each patient. These high-dose regions were visualized by taking 2D projections through the reference geometry in each principal anatomic plane (transverse, coronal, sagittal), producing images with pixel values representing the percentage of patients for whom the projected high-dose region was present at each point in the plane. Projections were convolved with a Gaussian smoothing kernel (3-mm standard deviation) to avoid identification of features smaller than the typical registration error. This process was carried out for both for the whole heart and for each cardiac substructure, allowing visual localization of the high-dose peak.

## Results

### Population, ECG, and radiation therapy dosimetry data

**Fig. 1:**
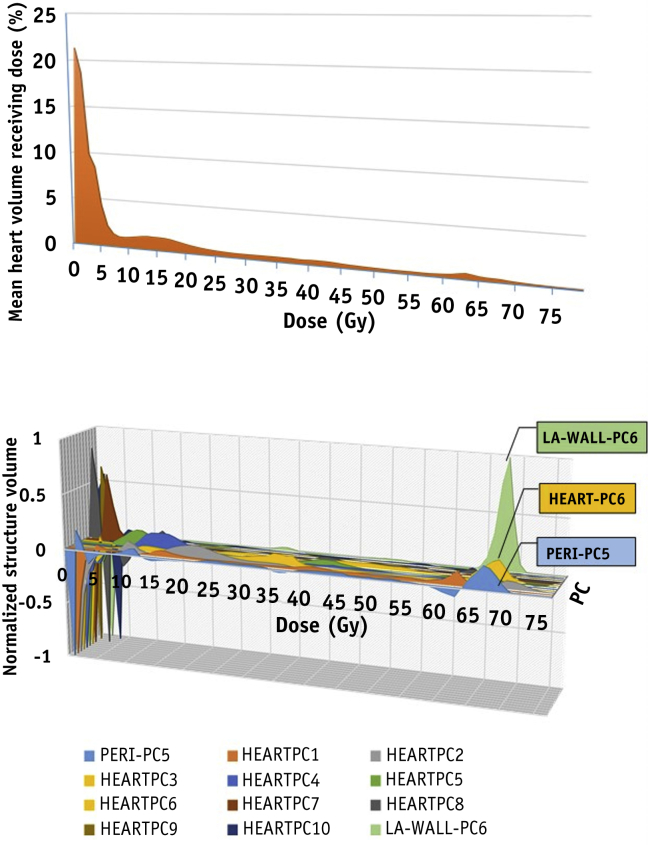
The mean whole-heart differential dose-volume histograms of 78 patients, plotted together with the 10 varimax-rotated principal components (PCs) describing >95% of the whole-heart dosimetric variance, and left atrial wall PC-6 (LA-Wall-PC6) and pericardium-PC5 (Peri-PC5). For ease of visualization, the loadings (fractional volumes receiving different dose levels) of the PCs have been scaled so that for each PC the maximum absolute value is 1.

Patient and treatment data are detailed in Table 1. ECG changes are also summarized in the table, together with whole-heart and cardiac substructure dosimetry. Fifty-three patients had analyzable ECGs both at baseline and 6 months after radiation therapy, and 20 patients (38%) had documented changes. The mean whole-heart dDVH is plotted in Figure 1, alongside the 10 varimax-rotated whole-heart PCs that described more than 95% of total variance.

**Table 1:** Treatment, ECG, and radiation dosimetry data for 78 patients analyzed

Characteristic	Value
Age, y, median (range)	66 (43-84)
WHO performance status∗, n (%)	
0	32 (41.0)
1	46 (59.0)
Sex, n (%)	
Female	20 (25.6)
Male	58 (74.4)
Stage, n (%)	
IIB	6 (7.7)
IIIA	54 (69.2)
IIIB	18 (23.1)
Nodal status, n (%)	
N 2 or 3	65 (83.3)
N 0 or 1	13 (16.7)
Histology, n (%)	
Squamous	42 (53.8)
Nonsquamous	36 (46.2)
PTV, cm^3^, median (range)	400.6 (138.7-1262.1)
Analyzable ECGs, n (%)	
Baseline	71 (91.0)
6 months	56 (71.8)
Baseline and 6 months	53 (67.9)
Normal ECGs, time point, n (%)	
Baseline	38 (48.7)
6 months	18 (23.1)
ECG changes from baseline to 6 months, n (%)	
Rhythm change	9 (11.5)
Ischemic or pericarditis-like change	11 (14.1)
Prescribed dose, Gy, median (range)	67.6 (63-73)
Mean EQD2†, Gy, median (range)	
Lung	14.7 (7.9-21.2)
Heart	8.0 (0.4-29.2)
Left atrium wall	17.1 (0.5-64.2)
Left ventricle wall	2.8 (0.3-26.9)
Right atrium wall	4.3 (0.3-61.3)
Right ventricle wall	3.8 (0.3-29.0)
Atrioventricular node	2.2 (0.3-52.5)
Pericardium	10.8 (0.4-27.0)

*Abbreviations:* ECG = electrocardiogram; PTV = planning target volume.

∗ Performance status 0–able to carry out all normal activity without restriction. Performance status 1–restricted in strenuous activity but ambulatory and able to carry out light work.

† EQD2: equivalent dose in 2 Gy fractions, calculated for α/β = 3 Gy.

### Associations between OS, clinical characteristics, ECG changes, and whole-heart dosimetry

Results from UVA are shown in Table 2. Patients with larger planning target volumes (PTVs) or “any ECG change” had significantly higher HRs for DR (*P*=.04 and .03, respectively). However, when analyzed separately, ischemic/pericarditis-like ECG changes and conduction ECG changes were not statistically significantly associated with DR. The presence of an abnormal baseline ECG was not significantly associated with DR (Table 2) or with onset of any ECG change at 6 months after treatment.

**Table 2:** Univariable Cox proportional hazards regression models of all-cause death rate versus clinical factors, whole heart dosimetry PCs, and cardiac substructure dosimetry PCs

Covariate	*P* value	Hazard ratio (95% confidence interval)
Clinical factors		
Baseline abnormal ECG	.96	0.98 (0.45-2.15)
Any ECG change at 6 months	.03	2.94 (1.12-7.76)
PTV size, cm^3^	.04	1.002 (1.000-1.003)
Prescribed dose, Gy	.25	0.93 (0.83-1.05)
Performance status, 0 vs 1	.35	1.49 (0.64-3.47)
Nodal stage, 0/1 vs 2/3	.42	1.64 (0.49-5.49)
Age, y	.60	1.01 (0.97-1.06)
Stage, IIba/IIIa vs IIIb	.80	1.13 (0.45-2.83)
Sex, female vs male	.82	1.11 (0.44-2.79)
Nonsquamous vs squamous	.94	1.03 (0.47-2.27)
Whole heart dosimetry		
Heart PC1	.28	1.18 (0.88-1.58)
Heart PC2	.12	0.52 (0.23-1.19)
Heart PC3	.56	0.83 (0.44-1.56)
Heart PC4	.77	0.94 (0.62-1.43)
Heart PC5	.48	1.15 (0.78-1.70)
Heart PC6	.01	1.54 (1.12-2.13)
Heart PC7	.78	1.05 (0.73-1.52)
Heart PC8	.60	1.11 (0.75-1.63)
Heart PC9	.16	0.73 (0.47-1.13)
Heart PC10	.65	0.92 (0.64-1.31)
Substructure dosimetry		
Left atrial wall PC6	.003	1.49 (1.14-1.95)
Pericardium PC5	.005	1.64 (1.16-2.33)

*Abbreviations:* ECG = electrocardiogram; PC = principal component; PTV = planning target volume.

Data from 78 patients were analyzed for all factors except ECG change, for which paired data were available for 53 patients. (*P* values are uncorrected for multiple hypothesis testing.)

Higher DR was significantly associated with larger Heart-PC6 scores (*P*=.009) (Table 2). Heart-PC6 has a prominent peak at 63 to 69 Gy and a dip at 0 to 4 Gy (Fig. 1); therefore, in patients with high Heart-PC6 scores, heart volumes receiving 63 to 69 Gy (V_Heart-63-69_) and 0 to 4 Gy (V_Heart-0-4_) are, respectively, relatively large and small. Higher DR is more plausibly linked to larger volumes receiving high doses than to smaller volumes receiving low doses—an interpretation supported by a significant association between V_Heart-63-69_ and DR (HR=1.13, *P*=.03) but not between V_Heart-0-4_ and DR (HR=1.002, *P*=.76). Heart-PC6 was not significantly correlated with PTV size (Pearson correlation *r*=.21; *P*=.06) or with prescribed dose (*r*=.08; *P*=.51) (Appendix E; available online at www.redjournal.org).

Significant correlations between DR and PTV size, “any ECG change,” and Heart-PC6 remained classified as positive discoveries when the false-discovery rate (FDR) was limited to 20% by the Benjamini-Hochberg procedure. The multivariable model judged best according to the AIC retained Heart-PC6 (*P*=.02), “any ECG change” (*P*=.04), and PTV size (*P*=.08) as factors associated with DR (Table 3) and had a good Harrell's C statistic of 0.76 (32).

**Table 3:** Multivariable Cox proportional hazards models of all-cause death rate judged best according to the AIC measure

Variable	*P* value	Hazard ratio (95% confidence interval)
Multivariable modeling including whole heart dosimetry^∗^ (Harrell's C statistic: 0.76)		
Heart PC6	.02	1.58 (1.08-2.33)
Any ECG change at 6 mo	.04	2.79 (1.03-7.50)
PTV size, cm^3^	.08	1.00 (1.00-1.01)
Multivariable modeling including whole heart and substructure dosimetry^†^ (Harrell's C statistic: 0.75)		
Left atrial wall PC6	.02	1.52 (1.07-2.17)
Any ECG change at 6 mo	.07	2.50 (0.92-6.81)
PTV size, cm^3^	.10	1.00 (1.00-1.01)

*Abbreviations:* AIC = Akaike information criterion; ECG = electrocardiogram; PC = principal component; PTV = planning target volume.

Factors initially included in the modeling were as follows: ^∗^clinical characteristics of Table 2 and whole-heart PCs with *P* value <.2 on univariable analysis (UVA) (PC2, PC6, PC9) and ^†^factors from * alongside substructure PCs with *P* value <.2 on UVA (Pericardium-PC5, Left atrial Wall-PC6). (*P* values are uncorrected for multiple hypothesis testing.)

### Associations between cardiac substructure dosimetry and OS

**Fig. 2:**
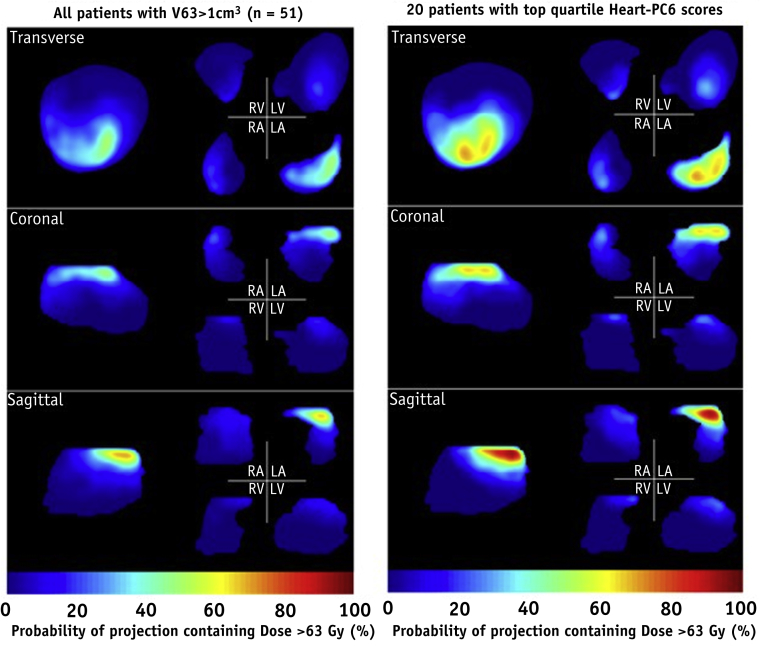
Three perspective views of the reference whole-heart geometry, and the left/right atria (LA/RA) and ventricles (LV/RV) plotted separately. The colorwash indicates percentages of patients whose heart dose distributions, mapped to the reference geometry, have values ≥63 Gy somewhere along lines perpendicular to the image plane. Data are shown for the 51 patients with V_Heart63_>1 cm^3^ (left) and the 20 patients with the highest Heart-PC6 scores (right).

The hearts of 62 patients received doses in excess of 63 Gy (the lower end of the peak of Heart PC6), and in 82% of these patients ≥1 cm^3^ of heart received ≥63 Gy. High dose levels were concentrated in the left atrium and overlying pericardium (Fig. 2): specifically, doses ≥63 Gy were delivered to the pericardium in all 62 patients, to LA-Wall in 48 (77%), RA-Wall in 22 (36%), LV-Wall in 18 (29%), RV-Wall in 16 (26%), and AVN in 2 (3%) patients. In the 20 patients with top quartile Heart-PC6 scores, the median heart and LA-Wall fractional volumes receiving ≥63 Gy (V_Heart63_, V_LA-wall63_) were 4% (range, 1%-18%) and 16% (range, 2%-77%), respectively, compared with 0.3% (range, 0%-14%) and 0% (range, 0%-22%) in the other 58 patients.

LA-Wall-PC6 and Pericardium-PC5 were the only substructure PCs with NDPs >1 (Appendix C; available online at www.redjournal.org). Like Heart-PC6, they both had prominent 63- to 69-Gy peaks (Fig. 1), and their scores were highly correlated with those of Heart-PC6 (*P*<.01, Spearman correlation coefficients of *r*=.81 and .91, respectively). LA-Wall-PC6 and Pericardium-PC5 scores were both significantly associated with DR on UVA (Table 2), and these associations remained classified as positive discoveries when the Benjamini-Hochberg procedure was used to limit the FDR to 20%, alongside the associations between DR and PTV size, “any ECG change,” and Heart-PC6 score.

In MVA of all factors including both whole-heart and substructure dosimetry, the model judged best according to the AIC retained higher LA-Wall-PC6 score (*P*=.02), presence of “any ECG change” (*P*=.07), and PTV size (*P*=.10) as factors associated with higher DR, the LA-Wall-PC6 score superseding Heart-PC6 (Table 3). This model's Harrell's C statistic was good at 0.75.

### Scale of OS variation with heart dosimetry and ECG changes

**Fig. 3:**
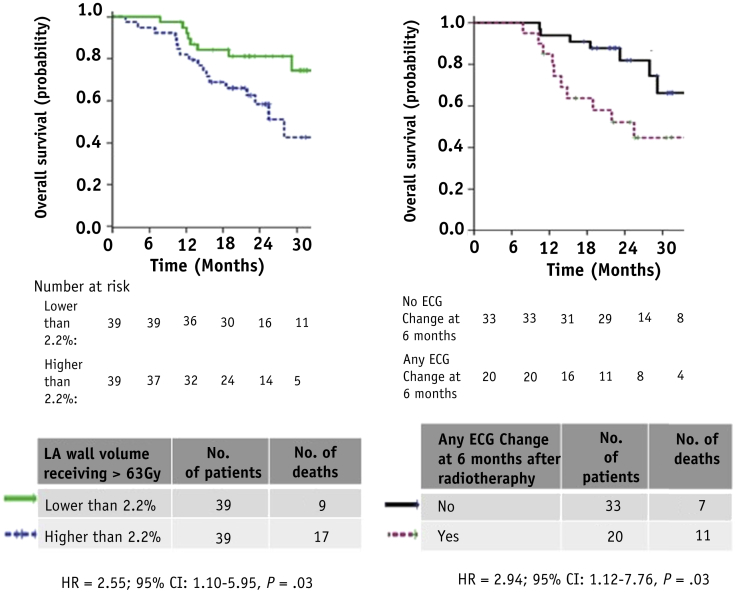
Kaplan-Meier overall survival (OS) curves for patients dichotomized by (left) volume of left atrial wall receiving >63 Gy (V_LA-Wall63_) being greater than/equal to or lesser than 2.2% (its median value) (78 patients, 26 events); (right) no electrocardiographic (ECG) change versus ECG change 6 months after treatment (53 patients, 18 events). *Abbreviations:* CI = confidence interval; HR = hazard ratio.

Table 2, Table 3 list DR hazard ratios for the factors studied on UVA and MVA. The scale of association between V_LA-Wall-63_ and DR can be seen in survival curves plotted in Figure 3: for patients with V_LA-Wall-63_ values below or above the median level of 2.2%, median OS was 39.2 and 27.9 months, respectively. At 24 months after treatment, survival was 23% higher (81% vs 58%) for patients with less highly irradiated atrial wall volumes. The scale of association between DR and ECG changes is shown in the survival curves split by the presence or absence of any ECG change, also plotted in Figure 3.

### Correlations between ECG changes and dosimetry

Correlations between “Any ECG change” and scores for Heart-PC6, Pericardium-PC5, and LA-Wall-PC6 were not significant (*P*=.90, .77, and .44, respectively) (Appendix G; available online at www.redjournal.org).

## Discussion

To our knowledge, this is the first study to use a DVH-wide analysis based on principal components to explore associations between heart irradiation and death rate in NSCLC patients, rather than using discrete dose-volume point metrics. It is also the first to analyze ECG changes and detailed cardiac substructure dosimetry in relation to DR in these patients.

We have found higher DR to be significantly associated with the presence of “any ECG change” 6 months after radiation therapy, and with large PTV size and larger values of V_Heart-63-69_, the whole-heart volume receiving 63 to 69 Gy. Cardiac doses of 63 to 69 Gy were concentrated in the left atrium and overlying pericardium, and correspond to 64 to 73 Gy equivalent dose in 2-Gy fractions (EQD2) for α/β = 3 Gy. The scale of associations between DR and heart and substructure volumes receiving 63 Gy was substantial, despite median values of these volumes being low (2%, 5%, and 8% for whole-heart, pericardium, and LA-Wall, respectively). Given that V_Heart-63-69_ was not significantly correlated with PTV size or prescribed dose, the association between heart dose and DR is likely independent of these latter 2 factors; indeed, both PTV size and Heart-PC6 are retained in the whole-heart multivariable model judged best by the AIC criterion, whereas prescribed dose is not significantly correlated with DR on univariable analysis.

We have additionally explored the impact on DR of the presence of N2/3 disease, and of subcarinal nodal involvement (Appendix H; available online at www.redjournal.org). On univariable analysis, neither N2/3 disease nor involvement of subcarinal nodes was significantly associated with DR (*P*=.42 and .12, respectively). After the addition of these factors into the multivariable models of Table 3, heart and left atrial wall PC6 remained significantly associated with DR (*P*=.03, with very slightly reduced HRs), whereas subcarinal nodal involvement was also retained in the multivariable model judged best on AIC (*P*=.07). In this dataset, then, there is no indication that nodal N2/3 disease or subcarinal nodal involvement was a confounder of the observed associations between cardiac dosimetry and DR.

Because of the proximity of the heart to lung tumors and nodes, it is difficult to avoid the entire heart when escalating tumor dose. It would therefore be useful to identify specific radiosensitive cardiac substructures to spare preferentially, but few data exist regarding cardiac substructure dosimetry. Although irradiation of the left atrial wall was significantly associated with DR in our study, it was not possible to determine whether this was because damage to this structure was particularly critical, or whether the association simply reflects the strong correlation between whole-heart and left atrial volumes receiving 63 to 69 Gy (Spearman correlation *r*=0.88, *P*<.01).

A recent review found no consensus on heart dose constraints used in NSCLC treatments, with most studies setting very lenient constraints especially at higher heart dose levels (33). In IDEAL-CRT, upper limits of 60, 53, and 45 Gy were placed on D_33%_, D_67%_, and D_100%_, respectively, the lowest doses delivered to the most highly irradiated 33%, 67%, and 100% of the heart (20). One study set a strict maximum heart dose limit of 63 Gy (34), and in light of the associations we have seen between DR and V_Heart63-69_ we are presently investigating whether this limit can be met while delivering the 63- to 73-Gy IDEAL-CRT range of prescribed doses. The lack of well-defined heart constraints reflects the scarcity of studies exploring the dose dependence of RIHD after radiation therapy for NSCLC.

Our results provide direct evidence that DR is associated with cardiac irradiation after radiation therapy for NSCLC. This adds to existing indirect evidence from the postoperative radiation therapy (PORT) meta-analysis, which demonstrated a 7% absolute reduction in 2-year OS and higher rates of non-cancer-related deaths for irradiated compared with nonirradiated NSCLC patients (35). Analysis of the SEER database found that this increased risk of death existed in patients treated with PORT between 1983 and 1988, but not between 1989 and 1993, possibly because of better normal tissue sparing after the introduction of conformal radiation therapy and higher-energy linear accelerators rather than cobalt machines (36). Greater heart toxicity and mortality rates have also been reported for NSCLC patients with irradiated left-sided rather than right-sided tumors 36, 37. Studies have found negative associations between OS and non-radiation therapy-related cardiac morbidity in NSCLC patients, suggesting that the additive effect of RIHD has the potential to further worsen OS 38, 39.

Radiation-induced fibrosis is thought to be a key mechanism in the development of cardiac dysfunction after radiation therapy. In rats, fibrosis accumulation and dose-dependent decreases in end-diastolic diameter were seen 1 month after single fractions of 15 or 20 Gy, and deaths due to cardiac failure occurred after 22.5 Gy 40, 41. The impact of atrial irradiation on survival was demonstrated by the higher survival seen when atria were specifically spared during heart irradiation (40).

Atrial fibrosis is commonly seen in patients with atrial fibrillation (AF) and plays an important role in the pathophysiology of AF 42, 43, 44, 45. Continuous AF can lead to LV dysfunction, increased LA pressure, and impaired atrial contractility, and it has been associated with worse clinical outcomes, including stroke (46). A surrogate for fibrosis, late gadolinium enhancement (LGE) on magnetic resonance imaging, has been seen in the left atrial walls of patients with AF. In patients with esophageal cancer, LGE has been seen in areas of heart receiving 40 Gy, and more so in regions receiving 60 Gy; however, it is not seen outside radiation fields (47). This dose effect is supported by evidence from an esophageal cancer study that found microvasculature circulation obstruction in 0%, 43%, and 68% of myocardial segments receiving 0 Gy, 40 Gy, and 60 Gy, respectively 48, 49. In another study, 14% of esophageal cancer patients had symptomatic cardiac disease 5 years after radiation therapy, the risk varying with the fractions of whole-heart volumes receiving ≥45 Gy, 50 Gy, and 55 Gy (50). In summary, imaging studies have demonstrated radiation dose–related cardiac changes in patients, but further work is required to directly associate these changes with morbidity and mortality.

Some limitations of our study must be acknowledged. First, although it was a prospective study, our analysis was post hoc and lacked comorbidity or smoking status data, which may be additive risk factors for RIHD. Second, the resting 12-lead ECG has limited value as an indicator of subtle pathologic changes within the heart 51, 52. However, in a similar population over the age of 65 without lung malignancies but with baseline ECG changes, the rate of nonfatal and fatal cardiovascular events at 6 months was only around 2% (53). Thus, the higher rate of ECG changes (38%) observed at 6 months in this study is likely due to the effect of radiation therapy.

Third, the study was relatively small, and its findings must consequently be interpreted with caution. Although we found DR to be associated with dosimetry and ECG changes, we could not distinguish truly cardiac-related deaths from cancer-related deaths because there was a lack of cardiac-specific morbidity or mortality data, and all patients with early death experienced tumor relapse. Recording of cause of death is complicated because deaths of radiation therapy patients after cardiac arrest could be scored as cancer related.

Fourth, although we found an association between high doses to left atrium and poorer survival, the mechanism remains unclear. Although we used a validated atlas (21) to define the cardiac substructure, there remains some uncertainty in our substructure outlines resulting from motion. Finally, although PCA provides a very efficient way of reducing many highly correlated dosimetric variables to a few factors, the clinical interpretation of the resulting varimax-rotated PCs can be challenging.

## Conclusion

In this small cohort of NSCLC patients we have seen significant associations between all-cause death rate and higher heart volumes receiving 63 to 69 Gy (particularly the left atrial wall). The observed associations suggest that small volumes of heart receiving high radiation doses may have a negative impact on survival, greater and more acute than that seen in breast cancer and lymphoma patients who are treated with lower radiation doses. Our study is hypothesis generating and requires further work to establish a causal relationship between radiation therapy and mortality. The pathophysiology of acute RIHD needs to be determined in prospective functional imaging studies before we recommend modification of treatment plans on the basis of these initial results, for example, to reduce heart doses receiving ≥63 Gy, particularly if this compromises dosimetric PTV coverage. Our finding of an association between DR and ECG changes at 6 months after treatment does, however, tentatively suggest a link between DR and RT-induced cardiac damage.

Given the armory of intensity modulated photon and ion beam radiation therapy technology available, outcomes of dose-escalated NSCLC treatments may be improvable in future by means of cardiac-sparing dose escalation techniques, given greater knowledge of links between survival, cardiac pathophysiology, and whole-heart and substructure dosimetry.

## Supplementary Data

Appendices A-H

## References

[1] Lim E., Baldwin D., Beckles M. (2010). Guidelines on the radical management of patients with lung cancer. Thorax.

[2] Perez C.A., Stanley K., Rubin P. (1980). A prospective randomized study of various irradiation doses and fractionation schedules in the treatment of inoperable non-oat-cell carcinoma of the lung. Preliminary report by the Radiation Therapy Oncology Group. Cancer.

[3] Perez C.A., Pajak T.F., Rubin P. (1987). Long-term observations of the patterns of failure in patients with unresectable non-oat cell carcinoma of the lung treated with definitive radiotherapy. Report by the Radiation Therapy Oncology Group. Cancer.

[4] Partridge M., Ramos M., Sardaro A. (2011). Dose escalation for non-small cell lung cancer: Analysis and modelling of published literature. Radiother Oncol.

[5] Socinski M.A., Blackstock A.W., Bogart J.A. (2008). Randomized phase II trial of induction chemotherapy followed by concurrent chemotherapy and dose-escalated thoracic conformal radiotherapy (74 Gy) in stage III non-small-cell lung cancer: CALGB 30105. J Clin Oncol.

[6] Kong F.M., Ten Haken R.K., Schipper M.J. (2005). High-dose radiation improved local tumor control and overall survival in patients with inoperable/unresectable non-small-cell lung cancer: Long-term results of a radiation dose escalation study. Int J Radiat Oncol Biol Phys.

[7] Kong F.M., Ten Haken R., Hayman J. (2011). Personalized high dose radiation (> 70 Gy) is significantly associated with better local regional control and overall survival in non-small cell lung cancer treated with concurrent chemoradiation. Int J Radiat Oncol Biol Phys.

[8] Stinchcombe T.E., Lee C.B., Moore D.T. (2008). Long-term follow-up of a phase I/II trial of dose escalating three-dimensional conformal thoracic radiation therapy with induction and concurrent carboplatin and paclitaxel in unresectable stage IIIA/B non-small cell lung cancer. J Thorac Oncol.

[9] Van Baardwijk A., Wanders S., Boersma L. (2010). Mature results of an individualized radiation dose prescription study based on normal tissue constraints in stages I to III non-small-cell lung cancer. J Clin Oncol.

[10] Bradley J.D., Moughan J., Graham M.V. (2010). A phase I/II radiation dose escalation study with concurrent chemotherapy for patients with inoperable stages I to III non-small-cell lung cancer: Phase I results of RTOG 0117. Int J Radiat Oncol Biol Phys.

[11] Machtay M., Bae K., Movsas B. (2012). Higher biologically effective dose of radiotherapy is associated with improved outcomes for locally advanced non-small cell lung carcinoma treated with chemoradiation: An analysis of the Radiation Therapy Oncology Group. Int J Radiat Oncol Biol Phys.

[12] van Baardwijk A., Reymen B., Wanders S. (2012). Mature results of a phase II trial on individualised accelerated radiotherapy based on normal tissue constraints in concurrent chemo-radiation for stage III non-small cell lung cancer. Eur J Cancer.

[13] Bradley J.D., Paulus R., Komaki R. (2015). Standard-dose versus high-dose conformal radiotherapy with concurrent and consolidation carboplatin plus paclitaxel with or without cetuximab for patients with stage IIIA or IIIB non-small-cell lung cancer (RTOG 0617): A randomised, two-by-two factorial phase 3 study. Lancet Oncol.

[14] Chun SG, Hu C, Choy H, et al. Comparison of 3-D conformal and intensity modulated radiation therapy outcomes for locally advanced non-small cell lung cancer in NRG Oncology/RTOG 0617. American Society for Radiation Oncology (ASTRO) 57^th^ Annual Meeting News Briefing, San Antonio, TX, October 18-21, 2015.

[15] Ramroth J., Cutter D.J., Darby S.C. (2016). Dose and fractionation in radiation therapy of curative intent for non-small cell lung cancer: Meta-analysis of randomized trials. Int J Radiat Oncol Biol Phys.

[16] Clarke M., Collins R., Darby S. (2005). Effects of radiotherapy and of differences in the extent of surgery for early breast cancer on local recurrence and 15-year survival: An overview of randomised trials. Lancet.

[17] Hancock S.L., Donaldson S.S., Hoppe R.T. (1993). Cardiac disease following treatment of Hodgkin's disease in children and adolescents. J Clin Oncol.

[18] Lin S.H., Wang L., Myles B. (2012). Propensity score-based comparison of long term outcomes with 3-dimensional conformal radiotherapy vs intensity modulated radiotherapy for esophageal cancer. Int J Radiat Oncol Biol Phys.

[19] Lin S.H., Zhang N., Godby J. (2016). Radiation modality use and cardiopulmonary mortality risk in elderly patients with esophageal cancer. Cancer.

[20] Landau D.B., Hughes L., Baker A. (2016). IDEAL-CRT: A Phase I/I trial of isotoxic dose-escalated radiotherapy and concurrent chemoradiotherapy in patients with stage II/III non-small cell lung cancer. Int J Radiat Oncol Biol Phys.

[21] Feng M., Moran J.M., Koelling T. (2011). Development and validation of a heart atlas to study cardiac exposure to radiation following treatment for breast cancer. Int J Radiat Oncol Biol Phys.

[22] Jackson J.E. (2003). A User's Guide to Principal Components.

[23] Dawson L.A., Biersack M., Lockwood G. (2005). Use of principal component analysis to evaluate the partial organ tolerance of normal tissue to radiation. Int J Radiat Oncol Biol Phys.

[24] Skala M., Rosewall T., Dawson L. (2007). Patient-assessed late toxicity rates and principal component analysis after image-guided radiation therapy for prostate cancer. Int J Radiat Oncol Biol Phys.

[25] Sohn M., Alber M., Yan D. (2007). Principal component analysis-based pattern analysis of dose-volume histograms and influence on rectal toxicity. Int J Radiat Oncol Biol Phys.

[26] Vespirini D., Sia M., Lockwood G. (2011). Role of principal component analysis in predicting toxicity in prostate cancer patients treated with hypofractionated intensity-modulated radiation therapy. Int J Radiat Oncol Biol Phys.

[27] Bauer J.D., Jackson A., Skwarchuk M. (2006). Principal component, Varimax rotation and cost analysis of volume effects in rectal bleeding in patients treated with 3D-CRT for prostate cancer. Phys Med Biol.

[28] Available at: http://www.statmethods.net/stats/regression.html. Accessed April 19, 2017.

[29] Akaike H. (1974). A new look at the statistical model identification. IEEE T Automat Contr.

[30] Harrell F.E., Lee K.L., Mark D.B. (1996). Multivariable prognostic models: Issues in developing models, evaluating assumptions and adequacy, and measuring and reducing errors. Stat Med.

[31] Pencina M.J., D'Agostino R.B. (2004). Overall C as a measure of discrimination in survival analysis: Model specific population value and confidence interval estimation. Stat Med.

[32] Available at: http://mchp-appserv.cpe.umanitoba.ca/viewDefinition.php?printer=Y&definitionID=104234.

[33] Fleming C., Cagney D.N., O'Keefe S. (2016). Normal tissue considerations and dose-volume constraints in the moderately hypofractionated treatment of non-small cell lung cancer. Radiother Oncol.

[34] RTOG 1106/ACRIN 6697: Randomized phase II trial of individualized adaptive radiotherapy using during treatment FDG-PET/CT and modern technology in locally advanced non-small cell lung cancer (NSCLC). Available at: https://www.rtog.org/ClinicalTrials/ProtocolTable/StudyDetails.aspx?study=1106.

[35] (1998). Postoperative radiotherapy in non-small-cell lung cancer: Systematic review and meta-analysis of individual patient data from nine randomised controlled trials. PORT Meta-analysis Trialists Group. Lancet.

[36] Lally B.E., Detterbeck F.C., Geiger A.M. (2007). The risk of death from heart disease in patients with nonsmall cell lung cancer who receive postoperative radiotherapy: Analysis of the Surveillance, Epidemiology, and End Results database. Cancer.

[37] Hardy D., Liu C.C., Cormier J.N. (2010). Cardiac toxicity in association with chemotherapy and radiation therapy in a large cohort of older patients with non-small-cell lung cancer. Ann Oncol.

[38] Islam K.M., Jiang X., Anggondowati T. (2015). Comorbidity and survival in lung cancer patients. Cancer Epidemiol Biomarkers Prev.

[39] Kravchenko J., Berry M., Arbeev K. (2015). Cardiovascular comorbidities and survival of lung cancer patients: Medicare data based analysis. Lung Cancer.

[40] Kitahara T., Liu K., Solanki K. (1993). Functional and morphological damage after local heart irradiation and/or adriamycin in Wistar rats. Radiat Oncol Investig.

[41] Krüse J.J., Zurcher C., Strootman E.G. (2001). Structural changes in the auricles of the rat heart after local ionizing irradiation. Radiother Oncol.

[42] Fukumoto K., Habibi M., Ipek E.G. (2016). Association of left atrial local conduction velocity with late gadolinium enhancement on cardiac magnetic resonance in patients with atrial fibrillation. Circ Arrhythm Electrophysiol.

[43] King J.H., Huang C.L., Fraser J.A. (2013). Determinants of myocardial conduction velocity: Implications for arrhythmogenesis. Front Physiol.

[44] Frustaci A., Chimenti C., Bellocci F. (1997). Histological substrate of atrial biopsies in patients with lone atrial fibrillation. Circulation.

[45] Burstein B., Nattel S. (2008). Atrial fibrosis: Mechanisms and clinical relevance in atrial fibrillation. J Am Coll Cardiol.

[46] Glotzer T.V., Daoud E.G., Wyse D.G. (2009). The relationship between daily atrial tachyarrhythmia burden from implantable device diagnostics and stroke risk: The TRENDS study. Circ Arrhythm Electrophysiol.

[47] Umezawa R., Ota H., Takanami K. (2014). MRI findings of radiation-induced myocardial damage in patients with oesophageal cancer. Clin Radiol.

[48] Umezawa R., Takase K., Jingu K. (2013). Evaluation of radiation-induced myocardial damage using iodine-123 β-methyl-iodophenyl pentadecanoic acid scintigraphy. J Radiat Res.

[49] Umezawa R., Takanami K., Kadoya N. (2015). Assessment of myocardial metabolic disorder associated with mediastinal radiotherapy for esophageal cancer: A pilot study. Radiat Oncol.

[50] Ogino I., Watanabe S., Iwahashi N. (2016). Symptomatic radiation-induced cardiac disease in long term survivors of esophageal cancer. Strahlenther Onkol.

[51] Mant J., Doust J., Roalfe A. (2009). Systematic review and individual patient data meta-analysis of diagnosis of heart failure, with modelling of implications of different diagnostic strategies in primary care. Health Technol Assess.

[52] Zimetbaum P.J., Josephson M.E. (2003). Use of the electrocardiogram in acute myocardial infarction. N Engl J Med.

[53] Jørgensen P.G., Jensen J.S., Marott J.L. (2014). Electrocardiographic changes improve risk prediction in asymptomatic persons age 65 years or above without cardiovascular disease. J Am Coll Cardiol.

