# Supplementary material for: The Impact of Cardiac Radiation Dosimetry on Survival After Radiation Therapy for Non-Small Cell Lung Cancer
Source: Int J Radiat Oncol Biol Phys. 2017 Sep 1;99(1):51–60. doi: 10.1016/j.ijrobp.2017.04.026 (PMC5554783; doi:10.1016/j.ijrobp.2017.04.026)
Supplement: Appendices A-H [file mmc1.pdf]

Supplementary Material

Appendix A – Approximating dDVHs using the mean dDVH and principal components

Denoting the dDVH of a structure in patient i as $dDVH_i$, and representing the fractional volume of the structure lying in the jth dose-bin as $dDVH_i(d_j)$, then dDVHi can be represented exactly as

$$\mathrm{dDVH_i}\left(\mathrm{d_j}\right) \approx \mathrm{mean\ dDVH}\left(\mathrm{d_j}\right) + \sum_k \mathrm{PC_{score-ki}} \times \mathrm{PC_k}\left(\mathrm{d_j}\right) \quad \text{(A1)}$$

where PC_k is the kth PC, $PC_{score\text{-}ki}$ is the score (contribution) of the kth PC for the dDVH of patient i, mean dDVH is the average of all patients' dDVHs for the structure, and k is summed over all PCs.

If k is only summed over a subset comprising the top-ranked PCs (which together account for a large fraction of the patient-patient dDVH variability), then (A1) will instead provide an efficient approximation to $dDVH_i$.

Appendix B – Benjamini-Hochberg procedure

The Benjamini-Hochberg (B-H) procedure is a technique for controlling the false discovery rate (FDR) following multiple hypothesis testing. It involves ranking the *p*-values of individual variables from smallest to largest, the smallest *p*-value having a rank of i=1, the next smallest i=2, etc. Each individual *p*-value is then compared to its B-H critical value, $(i/m)Q$, where i is the rank, m is the total number of tests, and Q is the level to which it is desired to limit the false discovery rate (FDR). The greatest *p*-value for which $p<(i/m)Q$ is significant, as are *all* of the *p*-values smaller than it, even any which individually are not lower than their B-H critical value. The FDR is

carefully chosen. Our work is a first exploratory step and requires further follow up studies to test the hypotheses generated. The cost of these additional studies is low (analysing heart dosimetry and OS data collected in the course of lung RT trials) and the cost of a false negative is high (missing a potentially important discover potentially impacting overall survival), and for these situations a fairly high FDR such as 0.20 is recommended.

1) Bejamini & Hochberg 1995, Journal of the Royal Statistical Society, Series B, Volume 57, No. 1, pp. 289-300.
2) http://www-01.ibm.com/support/docview.wss?uid=swg21476447

Appendix C – Alignment between substructure PCs and heart-PC$_{max}$

We define the normalized dot product (NDP) of a substructure PC (ss-PC) as

$$\mathrm{NDP} = \frac{\left(\sum_j \ \mathrm{ss{-}PC}(d_j) \times \mathrm{peak{-}PC}(d_j)\right) \ / \ \left\{\left(\sum_j \ \mathrm{ss{-}PC}(d_j)^2\right)\left(\sum_j \ \mathrm{peak{-}PC}(d_j)^2\right)\right\}^{1/2}}{\left(\sum_j \ \mathrm{heart{-}PC_{max}}(d_j) \times \mathrm{peak{-}PC}(d_j)\right) \ / \ \left\{\left(\sum_j \ \mathrm{heart{-}PC_{max}}(d_j)^2\right)\left(\sum_j \ \mathrm{peak{-}PC}(d_j)^2\right)\right\}^{1/2}} \quad \text{(C1)}$$

in which j indexes the dose-bins of each PC, and peak-PC is obtained from heart-PC$_{max}$ by setting all bins outside the high-dose peak of heart-PC$_{max}$ to zero. An NDP ≥1 indicates that the high-dose peak component of the substructure PC is at least as great as that of heart-PC$_{max}$.

The following table lists the normalised dot products for each of the cardiac substructure PC.

	Left atria	Left ventricle	Right atria	Right ventricle	AV node	Pericardium
PC1	0.306912162	0.099851359	0.205706513	0.028155981	0.382752649	0.149709752
PC2	-0.467627508	0.024479414	0.466217038	0.074305165	-0.029774186	0.039881623
PC3	-0.124375122	-0.004796849	-0.051173736	-0.002587203	0.003359913	0.193264718
PC4	-0.081010025	0.008979134	0.665452459	0.055443224	-0.012344046	0.013437174
PC5	-0.505477443	0.055721404	-0.084238845	0.005372654	0.002076228	1.19214625
PC6	2.702745151	-0.008113733	-0.0236041	-0.005795456	-0.001065049	-0.008349836
PC7	-0.56671751	-0.009068924	-0.012623973	-0.003294573	0.000143195	-0.083119054
PC8	-0.22824268	0.030478595	0.037079034	0.011040293	-0.000798996	-0.227436777
PC9	0.012507444	0.142853564	-0.006810306	0.008193253	0.000224774	0.056291724
PC10	0.037545323	-0.002099849	-0.172900165	0.02818046	0.000388441	0.011781409

Appendix D - Heart dose visualization

Data preparation

Radiotherapy planning data was exported from the treatment planning system in DICOM-RT format and converted into MATLAB (R2014a; Mathworks, Natick, MA) data-structures using functions from CERR (Computational Environment for Radiotherapy Research) [s1]. Binary label masks, indicating whether each voxel of the CT scan lies inside or outside the structure of interest, were extracted for the heart and cardiac substructures (atria and ventricles) of all patients. Planned dose distributions were interpolated on to the same grid as the CT scan and thresholded at ≥63 Gy, producing a binary mask of the volume which is dosimetrically similar to the previously-identified dDVH principal component.

Mask registration

One structure set was arbitrarily selected as a reference heart geometry, and a registration procedure was developed to find a coordinate transformation that maps each of the other hearts (the test hearts) on to the reference, as follows. Synthetic registration target images were created

from binary label masks for the test and reference hearts, with minor intensity variations introduced using the Euclidean distance transform from the surface of each substructure. An example image is presented in figure S1a). The MATLAB function imregtform was then used to identify the optimal affine transformation (translation, rotation, scale and shear) to map each test image to the reference image. The registration process used mean square difference as an image similarity metric, and a regular step gradient descent optimizer to perform a maximum of 100 iterations at 3 resolution levels. The resulting transformations were also applied to the thresholded dose distribution, allowing cross-comparison of the high dose volume on the geometry of the reference heart. The average final image following affine registration is presented in figure S1b).

Registration performance

Figure 2 illustrates the concordance of heart volumes in three dimensions before and after the registration process. The accuracy of the mapping was assessed quantitatively by comparing the masks for the 78 test hearts after transformation to the mask of the reference heart, using a series of standard metrics as given in table S1.

The Dice similarity value indicates that, on average, 90% of voxels are correctly mapped from within the test heart to within the reference heart. The mean distance to agreement between the surfaces of the test and reference hearts is typically less than 3mm, as is the average distance between their centres of mass.

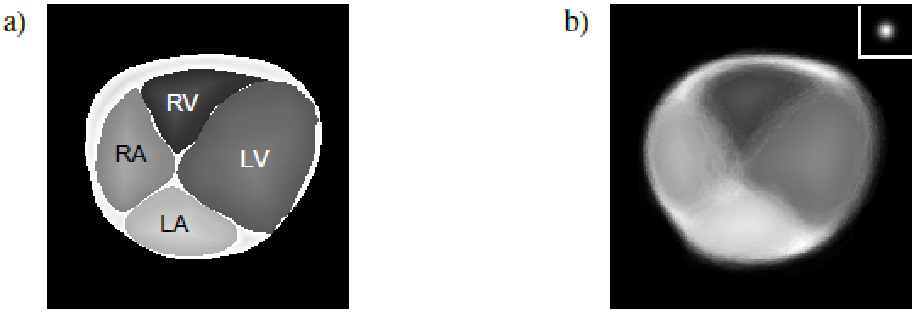

Figure S1: a) Synthetic registration target image for the reference heart, created from the delineated heart and substructures. Each substructure (labelled L/R = left/right, A/V = atrium/ventricle) is assigned an intensity, with individual voxel values modulated by the Euclidean distance transform from the substructure surface. The voxel value ranges are distinct for each substructure. Image show an axial slice through the centre of the heart. b) Average synthetic image over all test hearts after registration. Insert: Gaussian kernel (σ = 3mm) used to smooth final dose projections, to same scale as main image.

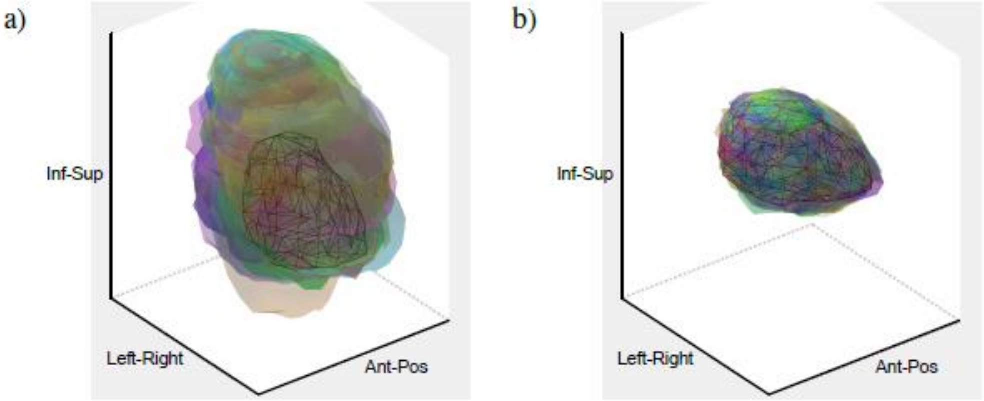

Figure S2: a) Simplified 3D renderings of all hearts in their original coordinate system before registration. Each heart is rendered in a different color. An example heart has been identified by black wireframe rendering. B) Equivalent 3D renderings following the registration process, with the same heart identified by black wireframe. Visual inspection suggests that for this heart the affine transformation constitutes at least translation, scaling and rotation.

Performance metric	Value (mean ± s.d.)
Dice similarity coefficient	0.90 ± 0.02
Jaccard similarity coefficient	0.83 ± 0.03
Mean distance to agreement	2.6 mm ± 0.6 mm
Centre of mass displacement	2.5 mm ± 0.7 mm

Table S1: Standard metrics used to assess the performance of the mask registration process

The overall performance of the process is considered acceptable, given that affine transformations cannot account for local deformation, although the results indicate that the method is not suitable for identifying features of order 3mm and smaller.

Visualization by projection

Two-dimensional projections were created from each of the registered high-dose volume masks in the coronal, sagittal and transverse planes. The process is equivalent to casting rays in the posterior-anterior, left-right or inferior-superior directions respectively: if a ray encounters one or more voxels of the mask, a ’1’ is recorded, and otherwise ’0’ is recorded. Projections in a given plane were summed for all hearts analyzed and the result was divided by the number of hearts, producing a 2D probability distribution for dose ≥63 Gy occurring at any point on the projection within the structure of interest. The resulting images were convolved with a Gaussian kernel of 3mm standard deviation (shown to scale in the inset of figure 1b) to avoid the identification of features smaller than the typical registration error, and are presented on the left-hand side of each panel in Figure 2 of the main text.

In order to produce the individual substructure images shown on the right-hand side of panels in Figure 2 of the main text, the high-dose mask was first restricted to the appropriate substructure using a Boolean AND operation. The process was otherwise identical.

References

[S1] J. O. Deasy, A. I. Blanco, V. H. Clark, CERR: A computational environment for radiotherapy research, Medical Physics 30 (5) (2003) 979–985. URL http://link.aip.org/link/?MPH/30/979/1

Appendix E – Correlations between Planning target volume (PTV), dose and heart-PC6 for all 78 patients analysed. (N=78)

		PTV	Dose	Heart_PC6
PTV	Pearson Correlation	1	-.36	.21
	Sig. (2-tailed)		.001	.06
dose	Pearson Correlation		1	.08
	Sig. (2-tailed)			.51
Heart_PC6	Pearson Correlation			1
	Sig. (2-tailed)			

Appendix F - Association between all-cause death-rate (DR) and HeartPC6 above or below mean value of 0 (high whole heart volume receiving 63-69Gy)

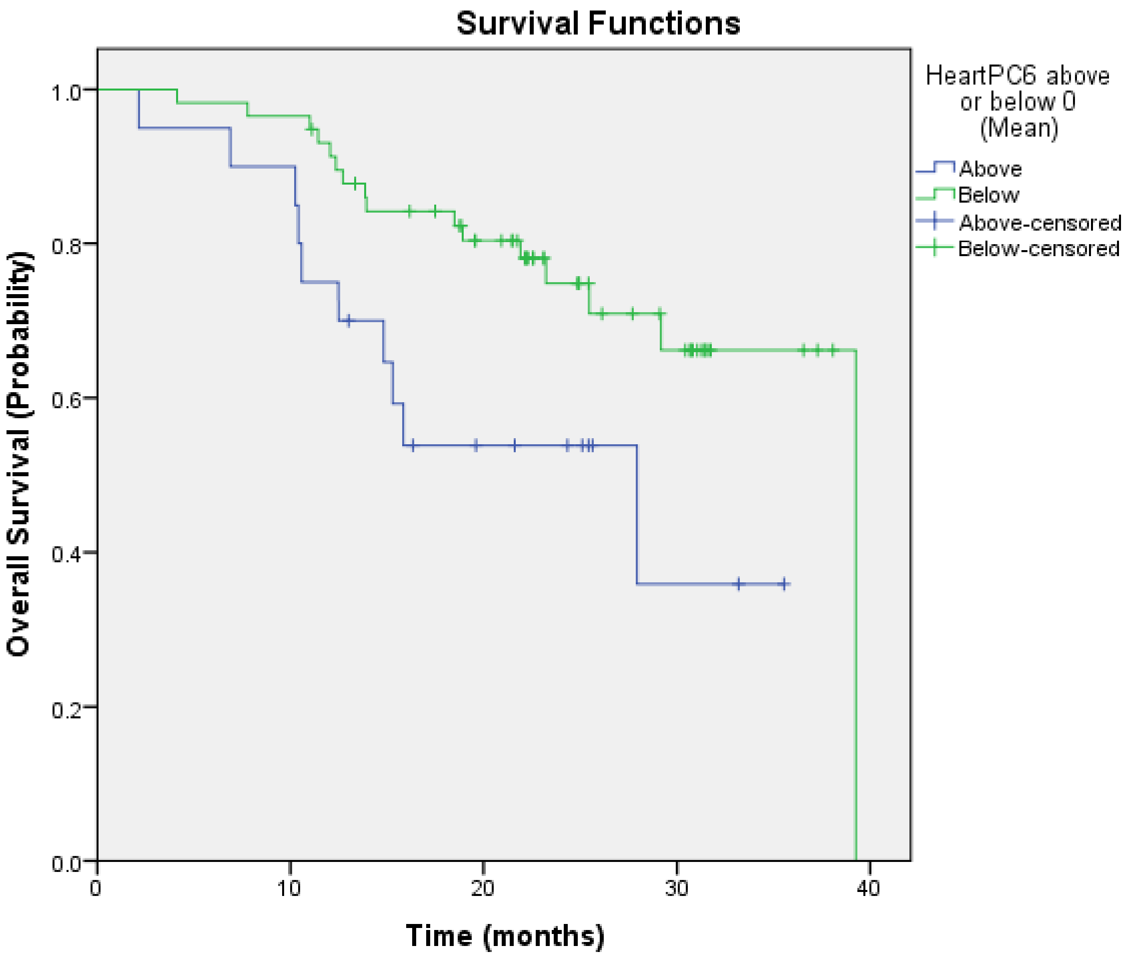

Log rank test p = 0.02

Appendix G - Univariable logistic regression analysis of Any ECG change versus dosimetric metrics at 6 months.

Co-variate	p-value	Odds ratio (95% confidence interval)
Heart-PC6	0.90	1.03 (0.62 - 1.73)
Pericardium-PC5	0.77	1.09 (0.63 - 1.86)
LA-Wall-PC6	0.44	1.23 (0.73 - 2.07)

Appendix H - Cox proportional hazards regression models of all-cause death-rate – effect of nodal stage and subcarinal nodal involvement

Univariable Cox proportional hazards regression models of all-cause death-rate versus nodal stage and subcarinal nodal involvement.

Univariable model	p-value	Hazard ratio (95% confidence interval)
**Clinical factors**		
**Nodal stage (0/1 vs 2/3)**	**.42**	**1.64 (0.49-5.49)**
**Subcarinal node**	**.12**	**1.92 (0.84-4.37)**

Multivariable Cox proportional hazards models of all-cause death-rate judged best according to the AIC measure. Factors initially included in the modelling were: * clinical characteristics of Table 2 (Replacing N2/3 stage with subcarinal nodal status) and whole-heart PCs with p-value

<0.2 on UVA (PC2, PC6, PC9); and $ substructure PCs with p-value <0.2 on UVA (Pericardium-PC5, Left atrial Wall-PC6) (p-values are uncorrected for multiple-hypothesis testing).

Multivariable modelling including whole heart dosimetry

Variables	p-value	Hazard ratio (95% confidence interval)
Heart-PC6	.027	1.56 (1.05-2.32)
Any ECG change at 6 months	.060	2.61 (0.96-7.09)
PTV size (cm^3)	.113	1.00 (1.00-1.00)
Subcarinal node	.072	2.54 (0.92-7.01)

Multivariable modelling including substructure dosimetry

Variables	p-value	Hazard ratio (95% confidence interval)
Left atrial Wall-PC6	.032	1.48 (1.03-2.12)
Any ECG change at 6 months	.087	2.41 (0.88-6.61)
PTV size (cm^3)	.119	1.00 (1.00-1.00)
Subcarinal node	.072	2.54 (0.92-7.02)
